# Longitudinal Trajectories of the Cognitive Function Index in the A4 Study

**DOI:** 10.14283/jpad.2024.125

**Published:** 2024-07-24

**Authors:** Rebecca E. Amariglio, J. D. Grill, D. M. Rentz, G. A. Marshall, M. C. Donohue, A. Liu, P. S. Aisen, R. A. Sperling, A4 Study team

**Affiliations:** 1 Department of Neurology, Mass General Brigham, Harvard Medical School, 60 Fenwood Road, Boston, MA 02115 USA; 2 Departments of Neurobiology and Behavior and Psychiatry, University of California Irvine School of Medicine, Irvine, CA 92697 USA; 3 Alzheimer’s Therapeutic Research Institute, Keck School of Medicine, University of Southern California, San Diego, CA 92121 USA; 4 https://www.actcinfo.org/a4-study-team-lists/

**Keywords:** Amyloid, positron emission tomography, preclinical Alzheimer’s disease, subjective cognitive decline

## Abstract

**Background:**

The Anti-Amyloid in Asymptomatic Alzheimer’s Disease (A4) Study failed to show a treatment benefit with solanezumab, but the longitudinal consequences of elevated amyloid were observed in study participants with objective decline on the Preclinical Alzheimer Cognitive Composite (PACC) and subjective decline on the combined Cognitive Function Index (participant + study partner CFI), during the trial period.

**Objectives:**

We sought to expand on previous findings by comparing longitudinal patterns of participant and study partner CFI separately and their associations with the PACC stratified by baseline amyloid tertile over the course of the A4 Study.

**Design:**

Cognitively unimpaired older adult participants and their study partners were independently administered the CFI at screen prior to amyloid PET disclosure and then at 3 subsequent visits (week 48, week 168, week 240) of the study. PACC collected at visits concurrent with CFI administration were also examined longitudinally.

**Setting:**

The A4 Study was conducted at 67 sites in Australia, Canada, Japan, and the United States.

**Participants:**

1,147 participants with elevated amyloid based on florbetapir PET were enrolled in the A4 Study and included in these analyses. 583 were on placebo and 564 were treated with solanezumab.

**Measurements:**

The PACC was used to assess objective cognitive performance and the CFI was used to assess change in everyday cognitive functioning by the participant and their study partner independently. Amyloid level was characterized by Centiloid tertiles (<46.1 CL, 46.1 to 77.2 CL, >77.2 CL). Participants were aware of their elevated amyloid status, but not their CL tertile, or specific level of amyloid. Longitudinal correlations between participant and study partner CFI and PACC were examined at all visits where assessments were available. The impact of baseline amyloid tertile on CFI and PACC associations was also examined.

**Results:**

Both participant and study partner CFI increased over the duration of the study indicating worsening cognitive functioning. Results did not differ by treatment group. The association between higher CFI and worse PACC for both for participant and study partner became progressively stronger over the course of the study. PACC had a significantly higher correlation with study partner CFI than with participant CFI by week 168. The stronger correlations between study partner CFI and PACC were driven by those in the highest amyloid tertile.

**Conclusion:**

Both participant and study partner report captured subtle changes in everyday cognitive functioning for participants with biomarker confirmed and disclosed preclinical AD. Moreover, study partner report was most highly aligned with cognitive decline, particularly among those with the highest amyloid load.

## Introduction

Clinical trials in symptomatic Alzheimer’s disease (AD) have historically required assessment of functional performance reported by a study partner, in addition to direct assessment of participant cognitive outcomes, to demonstrate a clinically meaningful therapeutic benefit (1). However, at the stage of preclinical AD (i.e., cognitively unimpaired but elevated amyloid), self-perceived changes in cognitive functioning are thought to most accurately reflect the everyday experiences of the participant compared to that from a study partner (2). What remains less clear, is the dynamic exchange between the accuracy of participant vs study partner report as participants become increasingly symptomatic, which can include reduced self-awareness. Previous studies comparing longitudinal trajectories of participant vs study partner report among those with elevated amyloid have shown that study partners report worsening cognitive functioning that is consistent with clinical progression, in contrast to participants whose report does not change significantly overtime, suggesting early signs of reduced self-awareness (3–5). While these studies have provided initial insights into the earliest symptoms of disease on longitudinal subjective report, previous studies did not typically disclose amyloid status to participants. As the field begins to deploy disease-modifying therapies, understanding the dynamic pattern between participant and study partner report, among those with biomarker confirmed and disclosed preclinical AD, is highly clinically relevant in tracking clinical progression and eventual treatment benefits.

The Anti-Amyloid in Asymptomatic Alzheimer’s Disease (A4) Study (6), was an early intervention trial targeting individuals at the stage of preclinical AD and testing solanezumab, a monoclonal antibody against soluble monomeric amyloid. While the treatment was ineffective, this study affords the unique opportunity to investigate change in outcomes among individuals with elevated amyloid who were unimpaired at baseline and aware of their biomarker status. For example, A4 participants and their study partners were annually administered the Cognitive Function Index (CFI) (7), a questionnaire that asks about change in cognitive functioning over the last year. Following screen, amyloid status was disclosed and enrolled participants and their study partners were administered the CFI annually for the duration of the study.

In this study, we sought to describe the longitudinal patterns of participant and study partner CFI and their associations with the Preclinical Alzheimer Cognitive Composite (PACC) (8), in participants enrolled in the A4 Study who all had elevated amyloid. We hypothesized that this association would be higher for study partner CFI by the end of the study compared to participant CFI based on previous work (9–10). While all participants had elevated amyloid, we also hypothesized associations between PACC and CFI would be most pronounced for study partner CFI in those with the highest levels of amyloid.

## Methods

A4 methods have been previously published (7), and we summarize the relevant aspects here as follows.

### Participant Data

Participants came from the modified intent-to-treat population of the A4 study, which consisted of 1,147 individuals, ages 65–85. For a detailed description of the screening procedures for the study please refer to (11). Briefly described here, participants were living independently without a diagnosis of Mild Cognitive Impairment (MCI) or dementia, and enrolled if they had a global Clinical Dementia Rating score of 0 (range 0–3), Mini Mental State Exam (12) score of 25–30 (range 0–30) and a Weschler Memory Scale-Revised (13) Logical Memory Delayed Recall score of 6–18 (range 0–25). Persons with unstable medical conditions were excluded, although participants with stable hypertension, diabetes, hypercholesterolemia, mild-to-moderate small-vessel ischemic disease and other medical conditions were eligible. Participants were randomized 1:1 to either placebo (n= 583) or solanezumab (n= 564). All participants received a baseline flobetapir PET scan, an annual CDR, and cognitive testing every six months with the Primary Alzheimer Cognitive Composite (PACC) (8) over the course of 4.5 years. All participants were required to have a study partner who was familiar with their functioning and consented to participate in data collection.

### Study Conduct and Brief Description of the Intervention

The A4 study was an early intervention trial that aimed to slow disease progression in clinically unimpaired (CU) older adults who had elevated amyloid on PET at baseline. The trial was conducted at 67 sites including Australia, Canada, Japan and the United States. Solanezumab is an immunoglobulin GI monoclonal antibody that binds to the mid-domain of the A-beta monomer (14). Eli Lilly provided the trial drug and placebo. Treatment was administered as a monthly infusion. The double-blind phase of the trial was conducted over 240 weeks with a double-blind extension to 312 weeks to accommodate those participants whose last visit was delayed due to the COVID 19 pandemic hiatus.

### Assessments

The PACC was the primary efficacy end point of the A4 trial (11). The PACC is comprised of the Free Recall plus Total (sum of free and cued) score from the Free and Cued Selective Reminding Test (15), the delayed paragraph recall on the Logical Memory IIa test from the Wechsler Memory Scale (13, 16), the Digit-Symbol Substitution Test from the Wechsler Adult Intelligence scale-Revised (17), and the Mini Mental Status Examination (MMSE) (12). Each component score was converted to a z-score by subtracting the baseline mean for that component and dividing by the baseline standard deviation for that component, resulting in the sum of four z-scores.

The CFI was originally developed as a mail-in screening instrument for AD prevention trials (7). The CFI is comprised of 15-items that capture change in cognitive functioning compared to 1-year prior (e.g., “Compared to one year ago, do you feel that your memory has declined substantially?” or “Compared to one year ago, do you have more difficulty managing money?”). The response to each question is score 0 (no), 0.5 (maybe), or 1 (yes) indicating whether there has been a change over the last year. Participants and their study partners completed the CFI independently. A total CFI score is calculated by adding up all the items from the participant and study partner versions of the questionnaire separately. In a prior study of longitudinal A4 data (6), the combined CFI was investigated, which is the sum of the participant CFI and the study partner report. In this study, we did not combine the participant and study partner CFI as we were interested in comparing their longitudinal patterns separately. The CFI was given at screen prior to amyloid disclosure. In subsequent visits (week 48, week 168 and week 240), participants and their study partners were administered the CFI knowing participants had elevated amyloid.

### Amyloid PET

Brain amyloid was assessed with 18F-Florbetapir PET using a mean cortical imaging standardized uptake value ratio (SUVR) with a cerebellar reference as previously described (7). An SUVR threshold of 1.15 identified individuals with early amyloid accumulation. An SUVR of 1.10 to <1.15 was considered elevated only when a visual read was considered positive by a two-reader consensus. SUVR’s were converted to centiloids on a scale of 0 to 100.

### Statistical Analyses

Natural cubic spline models were used to investigate the trajectory of the CFI (participant and study partner) by treatment group consistent with the primary analyses (6, 18). Models included effects for age, education, apolipoprotein E ε4 (APOE4) carrier status, baseline florbetapir SUVr (cortical amyloid aggregate), and two spline basis expansion terms for time per treatment group. Pearson’s correlation coefficients with 95% confidence intervals (CIs) were used to characterize association between PACC, participant CFI and study partner CFI at study visit time point. All variables were oriented so that higher scores indicated worse performance. We chose this approach since the analysis involved two continuous variables without outliers and assumed to be linearly associated within each time point. CIs for correlation differences (participant – study partner) were estimated using 1000 non-parametric bootstrap resamples. For analyses with amyloid tertile, natural cubic spline models were additionally used to examine CFI across amyloid tertiles adjusted for age, education, and APOE4 carrier status. When examining associations with PACC within tertile, a similar approach was employed with Pearson’s correlations and 95% CIs. Box-cox transforms were applied to evaluate the impact of the skewness on the final marginal estimate at week 240. All p-values and 95% CIs were nominal (i.e., not adjusted for multiplicity). All analyses were conducted using R version 4.4.0.

## Results

### Cohort characteristics

Baseline characteristics of the cohort have been previously published (6) and are presented in Table 1. To summarize, participants had a mean age of ∼72 years, had completed slightly > 16 years schooling, were 94% White, ∼ 60% female, had a mean baseline MMSE of ∼28, and 54.8% had at least 1 APOEε4 allele. The placebo and solanezumab groups were balanced on all baseline characteristics. There were no significant differences between treatment groups. Study partners were 64% spouses, 12.4% adult child or child-in-law, and 23.4% Other. They were 62% female with an average age of 65 years. 88% of participants retained their original study partner the entirety of the study.

**Table 1:** Baseline characteristics of the A4 cohort

	**Placebo (N=583)**	**Solanezumab (N=564)**	**Total (N=1147)**
Age (y)	71.9 (5.0)	72.0 (4.7)	72.0 (4.8)
Female sex	352 (60.4%)	329 (58.3%)	681 (59.4%)
Education (y)	16.6 (2.9)	16.6 (2.7)	16.6 (2.8)
Racial categories			
White	549 (94.2%)	531 (94.1%)	1080 (94.2%)
Black or African American	15 (2.6%)	12 (2.1%)	27 (2.4%)
Asian	13 (2.2%)	11 (2.0%)	24 (2.1%)
American Indian or Alaskan Native	0 (0.0%)	1 (0.2%)	1 (0.1%)
More than one race	3 (0.5%)	5 (0.9%)	8 (0.7%)
Unknown or Not Reported	3 (0.5%)	4 (0.7%)	7 (0.6%)
Ethnicity			
Not Hispanic or Latino	560 (96.1%)	542 (96.1%)	1102 (96.1%)
Hispanic or Latino	18 (3.1%)	16 (2.8%)	34 (3.0%)
Unknown or Not reported	5 (0.9%)	6 (1.1%)	11 (1.0%)
Family history of dementia (parent or sibling)	449 (77.0%)	411 (72.9%)	860 (75.0%)
APOEε4	342 (58.7%)	333 (59.0%)	675 (58.8%)
FBP SUVr	1.3 (0.2)	1.3 (0.2)	1.3 (0.2)
FBP Centiloid	65.9 (32.1)	66.2 (33.5)	66.0 (32.8)
PACC	−0.0 (2.6)	0.0 (2.7)	0.0 (2.7)
LM Delayed Recall	12.7 (3.5)	12.6 (3.8)	12.6 (3.7)
MMSE	28.8 (1.2)	28.8 (1.3)	28.8 (1.3)
CFI Participant	2.28 (2.1)	2.42 (2.2)	2.35 (2.2)
CFI Study Partner	1.35 (1.9)	1.62 (2.1)	1.48 (2.0)
GDS	1.0 (1.4)	1.1 (1.5)	1.1 (1.4)
CDR-Sum of Boxes	0.0 (0.2)	0.1 (0.2)	0.1 (0.2)

Summaries include means and standard deviations for continuous variables and counts with percentages for binary or categorical variables.

### Participant and study partner CFI by treatment group

**Figure 1a & 1b:**
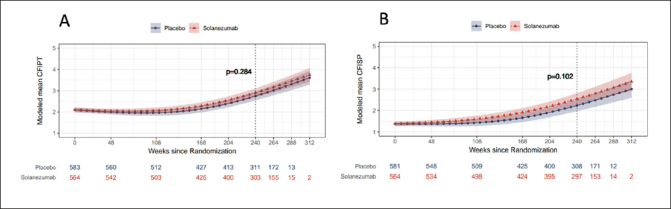
Natural Cubic Spline (NCS) modeled mean participant CFI (CFIPT) and study partner CFI (CFISP) The nominal p-value refers to treatment group contrast at 240 weeks.

Longitudinal PACC decline and combined CFI change has been previously reported, which did not differ statistically by treatment group (6). When examined separately, both participant and study partner CFI increased longitudinally over the course of the study indicating greater decline in cognitive functioning over time. There were no significant differences between placebo group and treatment group at week 240 in modeled mean participant CFI (mean difference 0.15, 95% CI −0.13 to 0.43) and study partner CFI (mean difference 0.31, 95% CI −0.06 to 0.67) (Figures 1a & 1b). Thus, the remaining analyses combined data from the two treatment groups.

### Participant and study partner CFI with PACC

**Figure 2:**
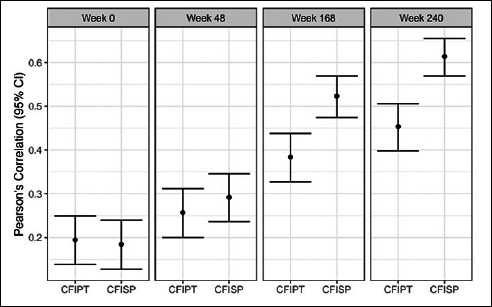
Pearson’s correlation between CFI Participant (CFIPT) and CFI Study Partner (CFISP) with PACC in both treatment groups combined All variables are oriented so that higher scores are for worse performance.

Correlations between both participant and study partner CFI and PACC increased in magnitude throughout the duration of the study (screen: correlation between PACC with participant CFI r=0.19 and study partner CFI r=0.18; week 240: correlation between PACC with participant CFI r= 0.45 and study partner CFI r= 0.61) (Figure 2) with higher CFI associated with worse PACC performance for all visits (screen average scores: PACC = 0.0, participant CFI = 2.35, study partner = 1.48; week 240 average scores: PACC= −1.02, participant CFI= 2.73, study partner CFI =2.09). When comparing the strength of the association between participant vs. study partner CFI with PACC there were no significant differences at screen (correlation difference 0.01, 95%CI: −0.06 to 0.08) or week 48 (correlation difference: −0.04, 95%CI −0.10 to 0.03), but for week 168 (correlation difference: −0.14, 95%CI −0.22 to −0.06) and week 240 (correlation difference: −0.16, 95%CI −0.23 to −0.09), study partner report was significantly stronger in its association with PACC compared to participant report.

### Participant and study partner CFI by amyloid tertile

**Figure 3 & 3b:**
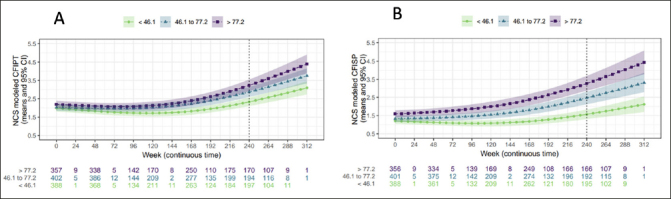
Natural cubic spline (NCS) modeled mean participant CFI (CFIPT) to the left and study partner CFI (CFISP) to the right CI −0.23 to −0.02), at week 168 (correlation difference: −0.16, 95% CI −0.28 to −0.04) and week 240 (correlation difference: −0.16, 95% CI −0.28 to −0.06).

Both participant and study partner CFI increased longitudinally across all amyloid tertiles (Figures 3a & 3b). Comparing across tertiles at week 240, there was a marginally significant difference in modeled mean participant CFI between low and middle tertiles (mean difference 0.54, 95%CI= 0.17 to 0.90), but not between the middle and high tertiles (mean difference 0.36, 95%CI= −0.06 to 0.79). By contrast, for study partner CFI, the difference between low and middle tertiles (mean difference 0.90, 95%CI= 0.46 to 1.34), as well as a between middle and high tertiles (mean difference 0.81, 95%CI= 0.27 to 1.36) were significant. There was a significant difference between the low and high tertiles for both participant (mean difference 0.90, 95%CI= 0.50 to 1.29) and study partner CFI (mean difference 1.71, 95%CI= 1.22 to 2.20).

When directly comparing participant vs study partner CFI by tertile at week 240 there was a significant difference between participant and study partner model mean score at the low tertile (mean participant CFI =2.34; mean study partner CFI=1.57; mean difference= −0.76, 95%CI −1.03 to −0.50) and middle tertile (mean participant CFI =2.87; mean study partner CFI =2.47; mean difference= −0.40, 95%CI −0.73 to −0.05), with the mean participant CFI score higher than study partner. However, there was no significant difference between participant and study partner CFI at the high tertile (mean participant CFI =3.25; mean study partner CFI=3.29; mean difference= −0.04, 95%CI −0.34 to 0.44).

### Participant and study partner CFI and PACC by amyloid tertile

**Figure 4:**
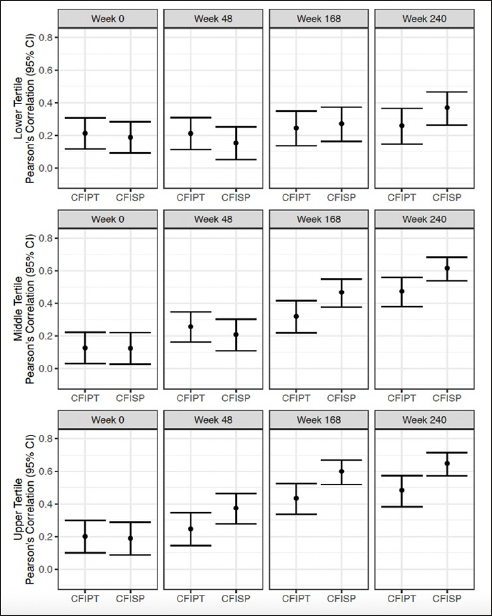
Pearson’s correlation between CFI Participant (CFIPT) and CFI Study Partner (CFISP) with PACC in both treatment groups combined All variables are oriented so that higher scores are for worse performance.

Correlations between participant CFI and PACC at week 240 were lowest for the low tertile (r =0.26) and increased for both the middle and high tertile (r= 0.47 and 0.48) (Figure 4). Correlations between study partner CFI and PACC at week 240, were also lowest for the low tertile (r= 0.37) and increased for the middle and high tertile (r: 0.62 and 0.65) (Figure 4).

When comparing correlation differences between participant and study partner CFI, there were no significant differences between participant and study partner report for the low tertile across all visits. For the middle tertile, there was a significant difference at week 168 (correlation difference: −0.15, 95% CI −0.29 to −0.01) and week 240 (correlation difference: −0.14, 95% CI −0.28 to −0.02). For the high tertile, there was a significant difference at week 48 (correlation difference: −0.13, 95%

## Discussion

Examining the CFI in the A4 Study provided the opportunity to characterize longitudinal participant vs study partner report in a trial of cognitively unimpaired participants with elevated amyloid. Importantly, subjective report was examined in the context of participants knowing their amyloid status, which was a unique aspect to this study. Previous studies have shown the likelihood of psychological harm after genetic or biomarker disclosure to cognitively unimpaired individuals is low (19, 20). However, knowledge of APOE4 carrier status and elevated amyloid PET scan results may lead to increased subjective report of cognitive concerns in the short-term (21, 22). This study was the first of its kind to examine longitudinal subjective report of cognitive functioning among those who knew they had elevated amyloid, but who did not know the severity of their amyloid load within this elevated range (i.e., amyloid tertile). By observing the greatest increase in CFI for those with very elevated amyloid, findings suggest that subjective report of cognitive functioning is associated with changes beyond a psychological reaction to knowing they are at risk for developing symptomatic AD.

When associating CFI with PACC, we consistently found that worse PACC performance was associated with higher CFI for both participant and study partner report at all visits. The strength of the association between CFI and PACC increased throughout the trial for both participant and study partner, likely due to greater variability in performance on both measures as time elapsed. Comparison of participant vs study partner CFI revealed that by week 168 the association between PACC and study partner CFI was stronger than participant CFI, consistent with prior findings that study partner CFI is more closely associated with PACC among those who demonstrate clinical progression (9, 10).

We next examined CFI trajectories with consideration of amyloid load. At the end of the study (week 240), we found that CFI endorsement was lowest in the low amyloid tertile compared to the high amyloid tertile for both participant and study partner CFI. When comparing adjacent tertiles, only low and middle tertiles were different for participant report (with no distinction between middle and high), whereas for study partner report for the CFI was significantly higher at each ascending amyloid tertile suggesting a noticeable delineation of disease severity with study partner CFI. These findings are in keeping with previous findings suggesting that at the very earliest stages of disease participant report tracks with subtle changes, but as individuals more closely approach symptomatic AD, participant report may not continue to increase due to reduced awareness (i.e., differentiation in CFI score only between low and middle tertile) (3–5).

Direct comparison between participant and study partner CFI by amyloid tertile did not reveal differences between participant and study partner CFI at the lowest tertile. However, in the highest tertile, differences in participant and study partner CFI were apparent by week 48, with study partner CFI score surpassing participant CFI score. Taken together, these findings make a strong case for the value of capturing study partner report in trials, even among participants with preclinical AD (23), as study partner report accelerated after approximately a year in those with the highest level of amyloid. This finding is consistent with prior longitudinal studies that show an increase in study partner report in those with elevated amyloid (3–5), but in this study we had the benefit of examining different amyloid levels within the elevated range, which allowed us to observe approximately how quickly (∼1 year) study partner report surpasses participant report at very high levels of amyloid.

Associations between PACC and CFI were also distinguishable among the amyloid tertiles. At week 240, correlations between PACC and CFI were lowest in the low tertile and increased in strength for the middle and high tertiles for both participant and study partner. When directly comparing participant and study partner CFI correlations with PACC, there were no correlation differences in the low tertile for participant and study partner report. By contrast, in the high tertile, associations between study partner CFI and PACC were significantly stronger than participant CFI by week 48, again suggesting how rapidly study partner report becomes more closely aligned with objective cognitive measures in those with very high amyloid.

### Limitations

Despite the important observations found in this study, there were several limitations. Without an effective treatment response from solanezumab, we were not able to determine whether an improvement on the CFI can be observed in the context of a successful treatment and how participant and study partner report might differ in such a setting. Additionally, this cohort was not representative of the larger older US population, as individuals had limited comorbidities, were highly educated and were mostly non-Hispanic White. All participants knew their elevated amyloid status and so we cannot rule out the possibility that the CFI scores were higher in every amyloid tertile group than it would have been in the absence of disclosure. However, we had the advantage of further examining differential patterns across amyloid tertiles, which was not shared with participants, providing evidence that findings were not observed solely on the basis of a reaction to amyloid disclosure or repeated testing exposure.

## Conclusions

Taken together, participant and study participant report of everyday cognitive functioning is highly valuable in tracking progression in those who have biomarker confirmed and disclosed preclinical AD. These findings are highly relevant to future prevention trials that will be selecting participants based on different thresholds of amyloid that aim to assess clinically meaningful change and eventually in the clinical context as individuals seek disease modifying therapies at the preclinical stage. Future work that more specifically delves into the temporal ordering of specific cognitive changes (i.e., item-level report) from participants and study partners longitudinally will additionally further characterize progression at the preclinical stage.

## Notes

*Ethical Standards:* Approval from an institutional review board was obtained at each of the sites and all participants and their study partners provided written informed consent prior to data collection.

## References

[1] FDA Early Alzheimer’s Disease: Developing Drugs for Treatment Guidance for Industry (draft March 2024).

[2] Jessen F, Amariglio RE, van Boxtel M, Breteler M, Ceccaldi M, Chételat G, et al. Subjective Cognitive Decline Initiative (SCD-I) Working Group. A conceptual framework for research on subjective cognitive decline in preclinical Alzheimer’s disease. Alzheimers Dement. 2014;10:844–52. doi: 10.1016/j.jalz.2014.01.001. Epub 2014 May 3. PMID: 24798886; PMCID: PMC4317324. 24798886 10.1016/j.jalz.2014.01.001 PMC4317324

[3] Dubbelman MA, Sikkes SAM, Ebenau JL, van Leeuwenstijn MSSA, Kroeze LA, Trieu C et al. Changes in self- and study partner-perceived cognitive functioning in relation to amyloid status and future clinical progression: Findings from the SCIENCe project. Alzheimers Dement. 2023 Jul;19:2933–2942. doi: 10.1002/alz.12931. Epub 2023 Jan 15. PMID: 36642977. 36642977 10.1002/alz.12931

[4] Hanseeuw BJ, Scott MR, Sikkes SAM, Properzi M, Gatchel JR, Salmon E et al. Evolution of anosognosia in alzheimer’s disease and its relationship to amyloid. Ann Neurol. Feb 2020;87:267–280. doi:10.1002/ana.25649 31750553 10.1002/ana.25649 PMC6980336

[5] Munro CE, Buckley R, Vannini P, DeMuro C, Sperling R, Rentz D et al. Longitudinal Trajectories of Participant- and Study Partner-Rated Cognitive Decline, in Relation to Alzheimer’s Disease Biomarkers and Mood Symptoms. Front Aging Neurosci. 2021;13:806432. doi:10.3389/fnagi.2021.806432 35173601 10.3389/fnagi.2021.806432 PMC8841868

[6] Sperling RA, Donohue MC, Raman R, Rafii MS, Johnson K, Masters CL et al. Trial of Solanezumab in Preclinical Alzheimer’s Disease. N Engl J Med. Sep 21 2023;389:1096–1107. doi:10.1056/NEJMoa2305032 37458272 10.1056/NEJMoa2305032 PMC10559996

[7] Walsh SP, Raman R, Jones KB, Aisen PS. Alzheimer’s Disease Cooperative Study Group. ADCS Prevention Instrument Project: the Mail-In Cognitive Function Screening Instrument (MCFSI). Alzheimer Dis Assoc Disord. 2006;20:S170–8. doi: 10.1097/01.wad.0000213879.55547.57. PMID: 17135810. 17135810 10.1097/01.wad.0000213879.55547.57

[8] Donohue MC, Sperling RA, Salmon DP, Rentz DM, Raman R, Thomas RG, et al. The preclinical Alzheimer cognitive composite: measuring amyloid-related decline. JAMA Neurol. 2014;71:961–70; 10.1001/jamaneurol.2014.803. 24886908 10.1001/jamaneurol.2014.803 PMC4439182

[9] Amariglio RE, Donohue MC, Marshall GA, Rentz D, Salmon D, Ferris S et al. Tracking early decline in cognitive function in older individuals at risk for Alzheimer disease dementia: the Alzheimer’s Disease Cooperative Study Cognitive Function Instrument. JAMA Neurol. Apr 2015;72:446–54. doi:10.1001/jamaneurol.2014.3375 25706191 10.1001/jamaneurol.2014.3375 PMC4397164

[10] Li C, Neugroschl J, Luo X, Zhu C, Aisen P, Ferris S et al. The Utility of the Cognitive Function Instrument (CFI) to Detect Cognitive Decline in Non-Demented Older Adults. J Alzheimers Dis. 2017;60(2):427–437. doi: 10.3233/JAD-161294. PMID: 28854503; PMCID: PMC6417419. 28854503 10.3233/JAD-161294 PMC6417419

[11] Sperling RA, Donohue MC, Raman R, Sun C-K, Yaari R, Holdridgw K et al. Association of Factors With Elevated Amyloid Burden in Clinically Normal Older Individuals. JAMA Neurol. Jun 01 2020;77:735–745. doi:10.1001/jamaneurol.2020.0387 32250387 10.1001/jamaneurol.2020.0387 PMC7136861

[12] Folstein MF, Folstein SE, McHugh PR. Mini-Mental State: A practical method for grading the cognitive state of patients for the clinician. Journal of Psychiatric Research. 1975;12:189–98 1202204 10.1016/0022-3956(75)90026-6

[13] Wechsler D. WMS-III, Wechsler Memory Scale-Third Edition, Administration and Scoring Manual. San Antonio, TX: The Psychological Corporation; 1997.

[14] Imbimbo BP, Ottonello S, Frisardi V, Solfrizzi V, Greco A, Seripa D, et al. Solanezumab for the treatment of mild-to-moderate Alzheimer’s disease. Expert Rev Clin Immunol. 2012;8:135–49; 10.1586/eci.11.93. 22288451 10.1586/eci.11.93

[15] Grober, E., C.B. Hall, R.B. Lipton, A.B. Zonderman, S.M. Resnick and C. Kawas, Memory impairment, executive dysfunction, and intellectual decline in preclinical Alzheimer’s disease. J Int Neuropsychol Soc, 2008. 14: 266–78 DOI: 10.1017/S1355617708080302. 18282324 10.1017/S1355617708080302 PMC2763488

[16] Morris, J., A. Swier-Vosnos, C. Woodworth, L.G. Umfleet, S. Czipri and B. Kopald, Development of alternate paragraphs for the Logical Memory subtest of the Wechsler Memory Scale-IV. Appl Neuropsychol Adult, 2014. 21(2): p. 143–7 DOI: 10.1080/09084282.2013.780172. 24826508 10.1080/09084282.2013.780172

[17] D, W., Wechsler Adult Intelligence Scale–Revised. 1981, San Antonio, TX: Psychological Corporation.

[18] Donohue MC, Langford O, Insel PS, van Dyck CH, Petersen RC, Craft S et al. Natural cubic splines for the analysis of Alzheimer’s clinical trials. Pharm Stat. 2023;22:508–519. doi:10.1002/pst.2285 36627206 10.1002/pst.2285 PMC10191992

[19] Lee AKW, Collier MK, Thompson LI, Popescu D, Arthur E, Correia S, et al. The Effects of Subjective Cognitive Decline on APOE Genotype Disclosure in the Butler Hospital Alzheimer’s Prevention Registry. J Prev Alzheimers Dis. 2023;10:152–161. doi: 10.14283/jpad.2023.12. PMID: 36946441. 36946441 10.14283/jpad.2023.12

[20] Grill JD, Raman R, Ernstrom K, Sultzer DL, Burns JM, Donohue MC, et al. Short-term Psychological Outcomes of Disclosing Amyloid Imaging Results to Research Participants Who Do Not Have Cognitive Impairment. JAMA Neurol. 2020 Dec 1;77:1504–1513. doi: 10.1001/jamaneurol.2020.2734. PMID: 32777010; PMCID: PMC7418046. 32777010 10.1001/jamaneurol.2020.2734 PMC7418046

[21] Lineweaver TT, Bondi MW, Galasko D, Salmon DP. Effect of knowledge of APOE genotype on subjective and objective memory performance in healthy older adults. Am J Psychiatry. 2014 Feb;171:201–8. doi: 10.1176/appi.ajp.2013.12121590. PMID: 24170170; PMCID: PMC4037144. 24170170 10.1176/appi.ajp.2013.12121590 PMC4037144

[22] Largent EA, Harkins K, van Dyck CH, Hachey S, Sankar P, Karlawish J. Cognitively unimpaired adults’ reactions to disclosure of amyloid PET scan results. PLoS One. 2020 Feb 13;15:e0229137. doi: 10.1371/journal.pone.0229137. PMID: 32053667; PMCID: PMC7018056. 32053667 10.1371/journal.pone.0229137 PMC7018056

[23] Grill JD, Karlawish J. Study partners should be required in preclinical Alzheimer’s disease trials. Alzheimers Res Ther. 2017 Dec 6;9:93. doi: 10.1186/s13195-017-0327-x. PMID: 29212555; PMCID: PMC5719524. 29212555 10.1186/s13195-017-0327-x PMC5719524

